# Caveolin-1: Functional Insights into Its Role in Muscarine- and Serotonin-Induced Smooth Muscle Constriction in Murine Airways

**DOI:** 10.3389/fphys.2017.00295

**Published:** 2017-05-15

**Authors:** Maryam Keshavarz, Heike Schwarz, Petra Hartmann, Silke Wiegand, Melanie Skill, Mike Althaus, Wolfgang Kummer, Gabriela Krasteva-Christ

**Affiliations:** ^1^Institute of Anatomy and Cell Biology, Justus-Liebig-University GiessenGiessen, Germany; ^2^Leibniz Institute for Prevention Research and Epidemiology - BIPSBremen, Germany; ^3^Institute of Anatomy and Cell Biology, School of Medicine, Saarland UniversityHomburg/Saar, Germany; ^4^German Center for Lung Research (DZL)Germany

**Keywords:** caveolin-1, airway smooth muscle, bronchus, contraction, muscarine, 5-HT

## Abstract

An increased bronchoconstrictor response is a hallmark in the progression of obstructive airway diseases. Acetylcholine and 5-hydroxytryptamine (5-HT, serotonin) are the major bronchoconstrictors. There is evidence that both cholinergic and serotonergic signaling in airway smooth muscle (ASM) involve caveolae. We hypothesized that caveolin-1 (cav-1), a structural protein of caveolae, plays an important regulatory role in ASM contraction. We analyzed airway contraction in different tracheal segments and extra- and intrapulmonary bronchi in cav-1 deficient (cav-1−/−) and wild-type mice using organ bath recordings and videomorphometry of methyl-beta-cyclodextrin (MCD) treated and non-treated precision-cut lung slices (PCLS). The presence of caveolae was investigated by electron microscopy. Receptor subtypes driving 5-HT-responses were studied by RT-PCR and videomorphometry after pharmacological inhibition with ketanserin. Cav-1 was present in tracheal epithelium and ASM. Muscarine induced a dose dependent contraction in all airway segments. A significantly higher Emax was observed in the caudal trachea. Although, caveolae abundancy was largely reduced in cav-1−/− mice, muscarine-induced airway contraction was maintained, albeit at diminished potency in the middle trachea, in the caudal trachea and in the bronchus without changes in the maximum efficacy. MCD-treatment of PLCS from cav-1−/− mice reduced cholinergic constriction by about 50%, indicating that cholesterol-rich plasma domains account for a substantial portion of the muscarine-induced bronchoconstriction. Notably, cav-1-deficiency fully abrogated 5-HT-induced contraction of extrapulmonary airways. In contrast, 5-HT-induced bronchoconstriction was fully maintained in cav-1-deficient intrapulmonary bronchi, but desensitization upon repetitive stimulation was enhanced. RT-PCR analysis revealed 5-HT1B, 5-HT2A, 5-HT6, and 5-HT7 receptors as the most prevalent subtypes in the airways. The 5-HT-induced-constriction in PCLS could be antagonized by ketanserin, a 5-HT2A receptor inhibitor. In conclusion, the role of cav-1, caveolae, and cholesterol-rich plasma domains in regulation of airway tone are highly agonist-specific and dependent on airway level. Cav-1 is indispensable for serotonergic contraction of extrapulmonary airways and modulates cholinergic constriction of the trachea and main bronchus. Thus, cav-1/caveolae shall be considered in settings such as bronchial hyperreactivity in common airway diseases and might provide an opportunity for modulation of the constrictor response.

## Introduction

Bronchoconstriction is a hallmark of asthma and COPD (Gosens et al., 2006). A major airway constrictor is acetylcholine (ACh), released from parasympathetic nerve fibers and acting upon muscarinic acetylcholine receptors (MR) types 2 and 3 on airway smooth muscle (ASM) (Long et al., 2009). In the course of inflammation, a wide range of additional contractile stimuli are released acting either directly on bronchial SM or indirectly through neural pathways leading to bronchial hyperresponsiveness (Buels et al., 2012; Scott and Fryer, 2012). The effects of serotonin (5-hydroxytryptamine, 5-HT) on ASM tone are complex, and both constrictor and relaxant actions have been reported, depending on species and concentrations used (Takahashi et al., 1995). In humans, bronchoconstriction is ascribed to 5-HT2A receptors and bronchodilation to 5-HT1A receptors on smooth muscle (Cazzola and Matera, 2000). Since free 5-HT levels in plasma are increased in asthma, it has been linked to pathophysiology of this disease (Lechin et al., 1996; Cazzola and Matera, 2000). 5-HT released from mast cells leads to contraction of ASM *in situ* and *in vitro* acting on a wide variety of G-protein-coupled 5-HT receptor subtypes in certain species (Ikawati et al., 2000; Kummer et al., 2006; Bai et al., 2007). The subtypes present in ASM appear to be species-dependent (Dupont et al., 1999).

There is evidence that both cholinergic and serotonergic signaling in ASM involves caveolae (Schlenz et al., 2010). Caveolae are flask-shaped plasma membrane invaginations containing high levels of cholesterol and glycosphingolipids that concentrate numerous structural proteins, ion channels, G-protein-coupled receptors and receptor kinases. They play a key role in numerous pathways associated with cell proliferation, migration and ASM constriction (Razani et al., 2002; Cohen et al., 2004; Ostrom and Insel, 2004; Gosens et al., 2007; Bastiani and Parton, 2010; Schlenz et al., 2010; Sharma et al., 2010). Expression of either caveolin-1 or −3 (cav-1 and −3, two caveolar proteins) is essential for caveolae formation and function (Bastiani and Parton, 2010). Cav-1 is widely expressed in type I pneumocytes, endothelial cells, adipocytes, fibroblasts and SM, whereas cav-3 is found primarily in striated (skeletal and cardiac) muscle and certain SM (Song et al., 1996; Razani et al., 2002). Caveolae and cav-1 appear to be of utmost importance in regulating the sensitivity of the ASM responses to ACh (Razani et al., 2002; Prakash et al., 2007; Gosens et al., 2011). In human ASM, cav-1 was identified as a marker of the contractile SM phenotype, and both, caveolae, and cav-1 are important for regulation of [Ca^+2^]_i_-mediated responses to agonists, as demonstrated by siRNA knockdown experiments (Prakash et al., 2007; Sharma et al., 2010; Gosens et al., 2011). Strikingly, the immunohistochemical analysis of endobronchial biopsies from asthmatic patients revealed a remarkable loss of cav-1 compared to the control group (Bains et al., 2012). In murine intrapulmonary airways, cholesterol depletion of the plasma membrane by methyl-β-cyclodextrin (MCD) markedly diminished the constrictor response to muscarine (Schlenz et al., 2010). Since murine bronchial SM express both, cav-1 and cav-3 (Schlenz et al., 2010), the relative contribution of these cav-isoforms to cholinergic bronchoconstriction could not be resolved by the MCD approach.

The same experimental paradigm (MCD-treatment) even fully abrogated the serotonergic constrictor response in murine intrapulmonary bronchi (Schlenz et al., 2010). Outside the airways, there is an evidence for the linkage of 5-HT receptors to caveolae and cav-1. For example, in vascular and gastrointestinal SM, the 5-HT2A receptor has been reported to be associated with caveolae (Dreja et al., 2002; Fiorica-Howells et al., 2002). A knockdown of cav-1 in C6 glioma cells nearly abolishes the 5-HT2A receptor-mediated signal transduction (Bhatnagar et al., 2004; Roth, 2011), and cav-1 regulates the levels of cell surface bound 5-HT7R in Hela cells (Sjögren and Svenningsson, 2007).

Hence, there is plenty of indirect evidence that cav-1 modulates muscarine- and 5-HT-induced airway constriction. We tested this hypothesis directly by studying airway constrictor responses in isolated extrapulmonary airways and intrapulmonary bronchi from cav-1 deficient (cav-1^−/−^) and corresponding wild-type (cav-1^+/+^) mice using organ bath recordings and videomorphometry of precision-cut lung slices (PCLS), respectively. In addition, MCD served as a tool for depletion of cholesterol from the membranes with the consequence of disruption of caveolar structure (Rodal et al., 1999) to discriminate between the specific role of the structural protein cav-1 and the general impact of caveolae and other cholesterol-rich membrane domains.

Taking into consideration that the composition of the airway wall and its reactivity to bronchoconstrictors change along the course of the conductive airways, we separately analyzed the sublaryngeal, middle and caudal, i.e., close to the bifurcation, trachea segments, and extra- and intrapulmonary bronchi. Since the receptor subtypes driving serotonergic airway responses in the mouse have not been fully elucidated yet, we investigated their expression by RT-PCR and used a selective inhibitor to determine the receptor subtype involved in 5-HT-induced SM constriction in intrapulmonary airways.

## Materials and methods

### Animals

Immunohistochemistry and functional experiments were performed on 12–22-week-old male cav-1^−/−^ (*n* = 33) mice and on the corresponding cav-1^+/+^ mice (*n* = 31) kept under specified pathogen-free (SPF) condition. The generation of cav-1^−/−^ mice with a genetic background of C57BL/6 × sv129 has been described previously (Drab et al., 2001; Razani et al., 2002). C57BL/6J mice (*n* = 4), 12–22 week-old, were used for studies of 5-HT-receptor subunits expression (RT-PCR). The animals were held according to the German guidelines for the care and use of laboratory animals. Animal experiments were approved by the local committee at the Regierungspräsidium Giessen, Hesse, Germany (permit No. GI 20/23-Nr. 60/2009; JLU-Nr. 450_GP; GI 20/23, JLU-Nt. 304). For videomorphometric analysis and immunohistochemistry, mice were killed by cervical dislocation. Otherwise, all animals were killed by inhalation of an overdose of isoflurane and exsanguination (Abbott, Wiesbaden, Germany).

### Substances

The following substances were used: MCD, muscarine, 5-HT and ketanserin were all purchased from Sigma-Aldrich, Munich, Germany. Muscarine, 5-HT and MCD were dissolved in water at 10 mM, ketanserin in DMSO at 50 mM, and diluted in HEPES to the desired experimental concentration immediately before use.

### Immunohistochemistry

Lungs and tracheae from cav-1^+/+^ (SPF, *n* = 3) and cav-1^−/−^ mice (SPF, *n* = 4) were filled with 50% Tissue-Tek (Sakura Finetek, Zoeterwoude, Netherlands) in 0.1 M phosphate buffer (pH 7.4), the thoracic viscera orientated on a piece of filter paper, and shock-frozen in cooled isopentane. Cryosections (10 μm) were cut, fixed with acetone at −20°C for 10 min, air-dried, and incubated for 1 h in 50% horse serum in PBS. The anti-cav-1 antibody, polyclonal from rabbit (dilution 1:400; sc-894; Santa Cruz Biotechnology, Heidelberg, Germany), was applied overnight at room temperature. After a washing step, the sections were incubated with Cy3-coupled donkey anti-rabbit IgG (1:2,000 in PBS; Chemicon, Hofheim, Germany) for 1 h at room temperature. The slides were rinsed, postfixed for 10 min in 4% paraformaldehyde (PFA), rinsed again, and coverslipped with carbonate-buffered glycerol (pH 8.6). The sections were evaluated with an epifluorescence microscope (Zeiss, Jena, Germany) using appropriate filter sets.

### Electron microscopy

PCLS from cav-1^+/+^ mice (SPF, *n* = 4) were collected after videomorphometric experiments (MCD-treated: *n* = 10, vehicle-treated: *n* = 9). Tracheae of cav-1^−/−^ (SPF, *n* = 6) and cav-1^+/+^ mice (SPF, *n* = 4) were prepared from animals whose vascular system was flushed via the ascending aorta with a rinsing solution containing heparin (2 ml/l; 10,000 U; Ratiopharm, Ulm, Germany), polyvinylpyrrolidone (25 g/l, MW 40,000; Roth, Karlsruhe, Germany) and procaine hydrochloride (5 g/l; Merck, Darmstadt, Germany) pH 7.4, followed by the fixative consisting of 1.5% glutardialdehyde and 2.5% PFA in 0.1 M phosphate buffer (pH 7.4). Dissected tracheae and PCLS were stored for additional 5 h in the same fixative, washed in 0.1 M TRIS-HCl buffer, osmicated for 2 h in aqueous 1% OsO_4_, washed (5 × 5 min) with water, stained overnight *en bloc* in 1% uranyl acetate in water, washed again (5 × 5 min) with water, dehydrated in ascending concentrations of ethanol and embedded in Epon. Sections of approximately 80 nm thickness were cut on an ultramicrotome (Reichert Ultracut E, Leica, Bensheim, Germany), stained with alkaline lead citrate, and examined under an EM 902 transmission electron microscope (Zeiss, Jena, Germany).

### Videomorphometry of intrapulmonary bronchi

Videomorphometric recordings from PCLS were performed as described previously (Schlenz et al., 2010). Briefly, the airways were filled via the cannulated trachea with low melting point agarose (Sigma, Taufkirchen, Germany). The lungs were dissected and cooled immediately. PCLS with 200 μm thickness were cut (vibratome VT1000S, Leica, Bensheim, Germany) from the left lobe of the lung and incubated in minimal essential medium (MEM, GIBCO, Karlsruhe, Germany) at 37°C under normoxic condition for 2–6 h to remove the agarose. The experiments were performed in a lung slice superfusion chamber (Hugo Sachs Elektronik, March, Germany) mounted on an inverted microscope. Images of bronchi of 150–250 μm in diameter were recorded with a CCD camera every 60 s and analyzed with Optimas 6.5 software (Stemmer Imaging, Puchheim, Germany). The bronchial luminal area was set as 100% after 5 min perfusion with HEPES-Ringer buffer. Bronchoconstriction and dilatation were expressed as a percentage decrease or increase of this area. Only those bronchi responding to 60 mM KCl with at least 20% reduction of their luminal area were included in the final data analysis. To address the possibility that 5-HT receptors might be sensitized or desensitized after prolonged or repeated application of 5-HT, we used different 5-HT application modes. The experimental designs were as follows:

- Exposure to KCl for 10 min, washing out for 20 min, exposure to additive ascending concentrations of muscarine (10 nM, 50 nM, 100 nM, 500 nM, 1 μM, 5 μM, 10 μM, 50 μM, 100 μM), each concentration for 10 min, followed by final washing out for 10 min.
- Exposure to KCl for 10 min, washing out for 20 min, exposure to additive ascending concentrations of 5-HT (100 nM, 500 nM, 1 μM, 5 μM, 10 μM, 50 μM, 100 μM, 500 μM, 1 mM), each concentration for 10 min, and final washing out for 10 min, application of KCl (viability control) for another 5 min.
- Exposure to KCl for 10 min, washing out for 20 min, exposure to additive ascending concentrations of 5-HT (10 nM, 100 nM, 1 μM, 10 μM, 100 μM, 1 mM), each concentration for 10 min, and final washing out for 10 min, application of KCl for another 5 min.
- Exposure to KCl for 10 min, washing out for 20 min, exposure to additive ascending concentrations of 5-HT (1 μM, 10 μM, 100 μM), each concentration for 10 min, and final washing out for 10 min, application of KCl for another 5 min.
- Exposure to KCl for 10 min, washing out for 20 min, exposure to additive ascending concentrations of 5-HT (100 μM, 1 mM), each concentration for 10 min, and final washing out for 10 min, application of KCl for another 5 min.
- Exposure to KCl for 10 min, washing out for 20 min, exposure to a single concentration of ketanserin (1 nM, 10 nM, 100 μM or 1 μM) for 10 min, exposure to ascending concentrations of 5-HT (10 nM, 100 nM, 1 μM, 10 μM, and 100 μM), each concentration for 10 min, and final washing out for 10 min, application of KCl for another 5 min.
- Stimulation with muscarine (1 μM) for 15 min, washing out for 15 min, stimulation with 5-HT (10 μM) for 10 min, washing out for 10 min and incubation with KCl for 5 min. Subsequently, the slices were washed again and incubated with 10 mM MCD or vehicle for 1 h at 37°C followed by restimulation with the same stimulants. Only those bronchi were included in the final data analysis that responded to stimulation with KCl in both recording series with reduction of their luminal area of at least 20%.

### Organ bath force recordings from trachea and extrapulmonary bronchi

The submandibular gland and the infrahyoid musculature were removed. The trachea was cut cranial to the larynx and caudal to the bifurcation and divided into 3 pieces, each consisting of 4 cartilage rings. The left extrapulmonary bronchus was dissected separately. Connective tissue and blood vessels were removed. Isometric contraction was measured in isolated rings that were mounted between two stainless steel clips in vertical 15 ml organ baths of a computerized isolated organ bath system (ADInstruments GmbH, Heidelberg, Germany).

The chamber was filled with 37°C warm MEM-Medium (Invitrogen Gibco, Oslo, Norway), which was supplemented with 1% penicillin/streptomycin (PAA Laboratories GmbH, Coelbe, Germany) and continuously aerated with a 95% O_2_/5% CO_2_ gas mixture. The temperature was held at 37°C by the use of a bath circulator (Thermo Fisher Scientific, Waltham, USA). The upper stainless clip was connected to an isometric force transducer (Power Lab 8.30; ADInstruments GmbH, Heidelberg, Germany). Tissues were equilibrated against a passive load of 1 g for all rings. After this period, samples were adjusted at 0.5 g tension. Changes in the isometric contraction were converted by the transducer into an amplified DC output voltage and assigned to the software LabChart 6 (ADInstruments GmbH, Heidelberg, Germany).

All samples were equilibrated for 30 min until they reached a stable baseline tension. At the beginning of each experiment, 60 mM KCl was administered to estimate the reference response. Rings were then washed with fresh MEM solution. After the tension returned to baseline, cumulative administration of ascending doses of muscarine and 5-HT was conducted. Changes in tension were recorded as force [measured in grams] and evaluated by software. The experimental designs were as follows:

- Exposure to KCl for 5 min, washing out for 20 min, exposure to additive ascending concentrations of muscarine (1 nM, 5 nM, 10 nM, 50 nM, 100 nM, 500 nM, 1 μM, 5 μM, 10 μM, 50 μM), each concentration for 10 min, and final washing out for 10 min. The value recorded 1 min directly before KCl stimulus application was considered as the baseline and set to 100%. The responses to different stimuli were calculated as percentage of baseline.
- Exposure to KCl for 5 min, washing out for 20 min, exposure to additive ascending concentrations of 5-HT (10 nM, 50 nM, 100 nM, 500 nM, 1 μM, 5 μM, 10 μM, 50 μM, 100 μM), each concentration for 10 min, final washing out for 10 min and application of KCl for another 5 min.

Each type of experiment was performed on samples from at least 5 animals; the exact number of animals is indicated in the graphs.

KCl was used for estimation of the receptor-independent contraction. The reactivity of the response to muscarine or 5-HT was calculated as follows:

$$\begin{array}{l} \text{Reactivity}[\text{muscarine}]\text{​}=\text{​Max force }[\text{muscarine}]/\text{Max force}[\text{KCl}]\\ \text{ or}\\ \text{ Reactivity}[\text{5}-\text{HT}]=\text{Max force}[\text{5}-\text{HT}]/\text{Max force }[\text{KCl}]. \end{array}$$

### RT-PCR

Tracheal muscle, left bronchus and pieces of peripheral lung were obtained from C57BL/6J mice (all *n* = 4). The samples were shock-frozen in RLT buffer plus (Qiagen, Hilden, Germany) and stored at −80°C until use. Total RNA was isolated by using the RNeasy method according to the manufacturer's protocol (Qiagen). The contaminating DNA was degraded using 1 U DNase-I (Invitrogen, Karlsruhe, Germany) per μg of total RNA, and a reverse transcription was done for 50 min at 42°C using 200 U Superscript II reverse transcriptase (Invitrogen) per μg of RNA. RT-PCR was performed by adding 1 μl cDNA, 0.5 μl of each intron-spanning primer (20 pM; MWG Biotech, Ebersberg, Germany) for β-2-microglobulin (serving as efficacy control) and for all 5-HT receptor subtypes (Table 1), 2.5 μl 10 × PCR buffer II (100 mM Tris-HCl, 500 mM KCl, pH 8.3), 2 μl MgCl_2_ (15 mM), 0.5 μl dNTP (10 mM each), 0.1 μl AmpliTaq Gold polymerase (5 U/μl; all reagents from Applied Biosystems, Darmstadt, Germany) and 17.9 μl H_2_O. The cycling conditions were 12 min at 95°C, 40 cycles with 30 s at 95°C, 30 s at 59°C, 30 s at 72°C, and a final extension at 72°C for 7 min. Control reactions included the absence of DNA template and the absence of reverse transcriptase. A 100 bp DNA ladder (Invitrogen, Karlsruhe, Germany) was run as marker (6.5 μl). The PCR products were separated by electrophoresis on a 2% TRIS-acetate-EDTA agarose gel and were detected by UV illumination using a spectrophotometer (Ultrospec 2100 Pro, Biochrom, Cambridge, UK).

**Table 1:** **Oligonucleotide primer sequences used in RT-PCR analysis**.

**Gene**	**Genebank accession no**.	**Primer**	**Product length (bp)**
5-HT1A R.	NM008308.4	fwd tctctccctccctcttgctc rev aattccagggcaccataacc	133
5-HT1B R.	NM010482	fwd aagccaaagcagaggaggag rev cggtcttgttgggtgtctgt	177
5-HT1D R.	NM008309	fwd cacggcacagcttatcacag rev cccagggtcttagtggcttt	192
5-HT1F R.	NM008310	fwd tggcattgaactgtgaatgg rev agaattttggatggcattcg	207
5-HT2A R.	NM172812.1	fw atagccgcttcaactccaga rev tcatcctgtagcccgaagac	106
5-HT2BR.	NM008311.2	fwd gggctactgcattcatcaaga rev ctcacaggtgacattgtgtgg	119
5-HT2C R.	NM008312.4	fwd gttcaattcgcggactaagg rev tcacgaacactttgctttcg	116
5-HT3A R.	NM013561.2	Fwd catgtatgccatcctcaacg rev ccacgtccacaaactcattg	188
5-HT3B R.	NM020274.4	fwd ggcagcttctttctgtgtcc rev gaagaccgtatagcccacca	233
5-HT4 R.	NM008313.4	fwd ttaatgttgggaggctgctg rev gggcttgttgaccatgaaga	156
5-HT6 R.	NM021358.2	fwd ggtgccatctgcttcaccta rev gcagccaggtgacaaagaac	250
5-HT7 R.	NM008315	fwd gccacttcttctgcaacgtc rev ttcacattctgagcccatcc	226
ß-2-Microglobulin	NM009735	fwd attcacccccactgagactg rev gctatttctttctgcgtgcat	192

### Statistical analysis

Data in graphs depicting time courses or dose responses are presented as mean ± standard error of mean (SEM) was used. Statistical analyses were performed using GraphPad Prism software, version 7 (La Jolla, CA, USA). All datasets were analyzed for normal distribution using Kolmogorov-Smirnov normality test. Normally distributed data were analyzed using parametric tests (Student's unpaired *t*-test or One-Way ANOVA followed by Tukey's multiple comparisons test), whereas data which failed the normality test was analyzed by non-parametric tests (Mann-Whitney U-test or Kruskall-Wallis test followed by Dunn's multiple comparisons test). Values for maximal efficacy (E_max_) and pEC_50_ values of agonist responses were estimated using non-linear regression sigmoidal curve analysis according to the Hill equation. The pA2 value for ketanserin was estimated by Schild-analysis. Interaction between treatments of mice strains were analyzed by 2-way ANOVA. Differences were considered as statistically significant when *p* ≤ 0.05.

## Results

### Cav-1 and caveolae presence in tracheal and bronchial SM

**Figure 1:**
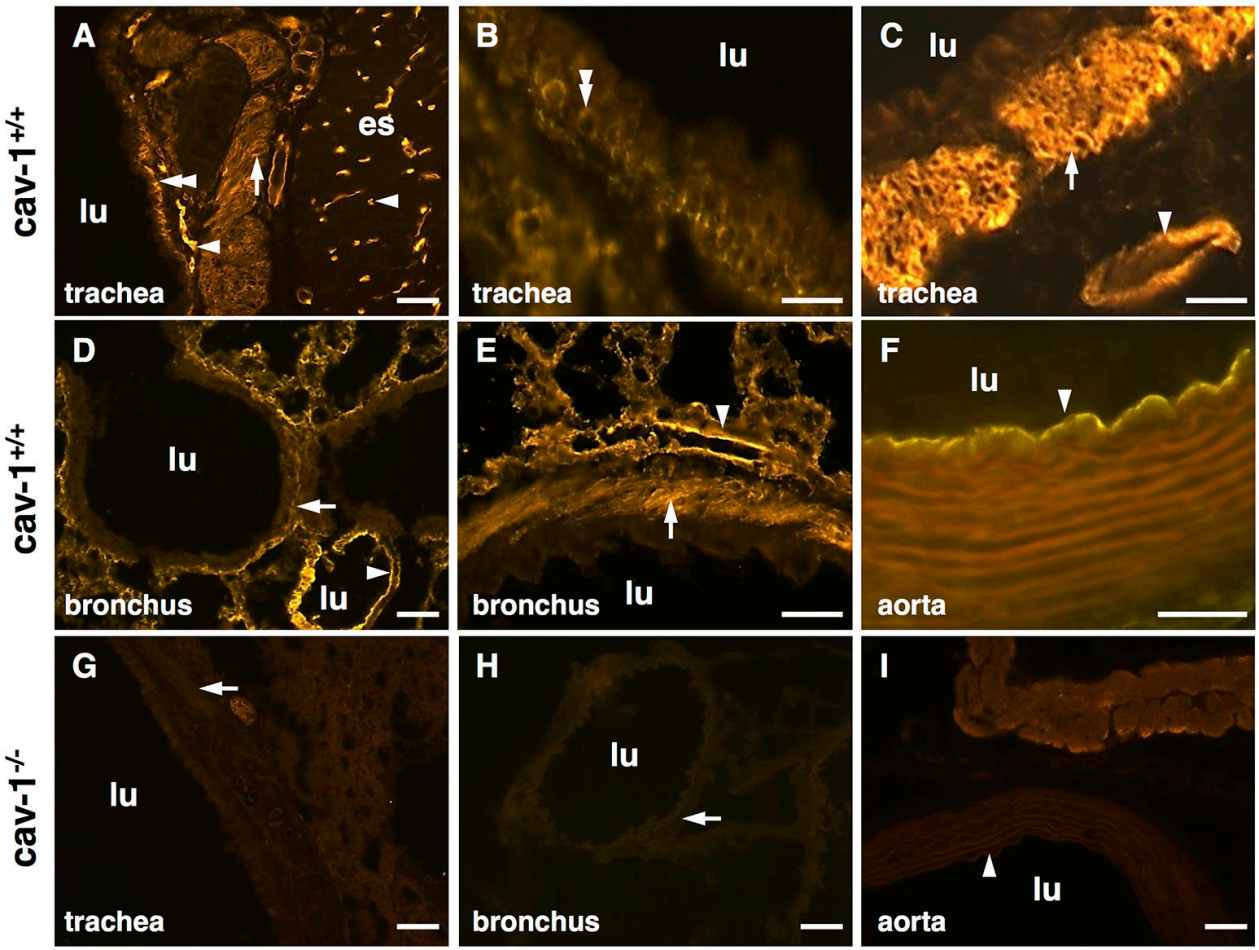
**Localization of cav-1 in murine thoracic organs using immunohistochemistry**. Images are representative for cav-1^+/+^ (*n* = 3 animals) and cav-1^−/−^ mice (*n* = 4 animals). **(A–C)** Cav-1-immunoreactivity is observed in endothelial cells of the esophagus and trachea and in tracheal basal epithelium and both tracheal and bronchial SM. **(D,E)** Alveolar region of the lung, bronchial SM and cells present in the alveolar wall (endothelial cells, epithelial cells) are immunoreactive for cav-1. **(F)** Aorta. **(G–I)** Controls for the specificity of the anti-cav-1 antibody. The cav-1-labeling is not present in cav-1^−/−^ trachea, lung and aorta. Arrowhead, endothelial cell; double arrowhead, basal epithelium; arrow, SM; lu, lumen; es, esophagus; bar, 50 μm.

**Figure 2:**
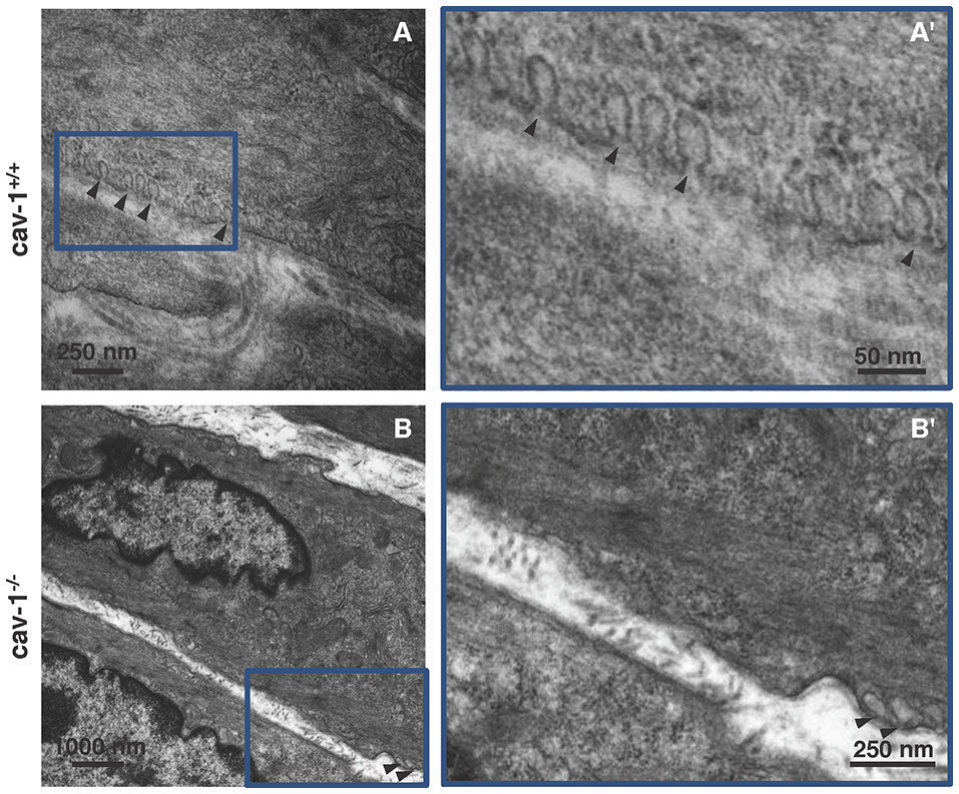
**Ultrastructure of smooth muscle from cav-1^+/+^ and cav-1^−/−^ mice**. Transmission electron microscopy. Images are representative for tracheae of cav-1^−/−^ (*n* = 6 animals) and cav-1^+/+^ mice (*n* = 4 animals). **(A,A')** Cav-1^+/+^ mice. Tracheal smooth muscle cells exhibit numerous caveolae that are located in groups at the plasma membrane. **(B,B')** Cav-1^−/−^ mice. Tracheal smooth muscle cells have low number of caveolae (arrowheads) at the plasma membrane. Caveolae = arrowhead.

Cav-1-immunoreactivity was detected in the tracheal epithelium, and in tracheal and bronchial SM (Figures 1A–E). A strong cav-1-immunoreactivity was observed in capillary endothelial cells in the esophagus (Figure 1A), in the trachea (Figure 1C), in the lung (Figures 1D,E) and in the aorta which served as positive control tissues (Figure 1F). The specificity of the anti-cav-1 antibody was confirmed by the absence of cav-1-immunolabeling in cav-1^−/−^ mice (Figures 1G–I). In agreement with these results, we observed numerous caveolae at the plasma membrane of tracheal SM from cav-1^+/+^ mice (Figure 2A) using electron microscopy. A substantial, but not complete loss of caveolae was observed in cav-1^−/−^ mice (Figure 2B).

### Muscarine-induced contraction in extra-pulmonary airways

**Figure 3:**
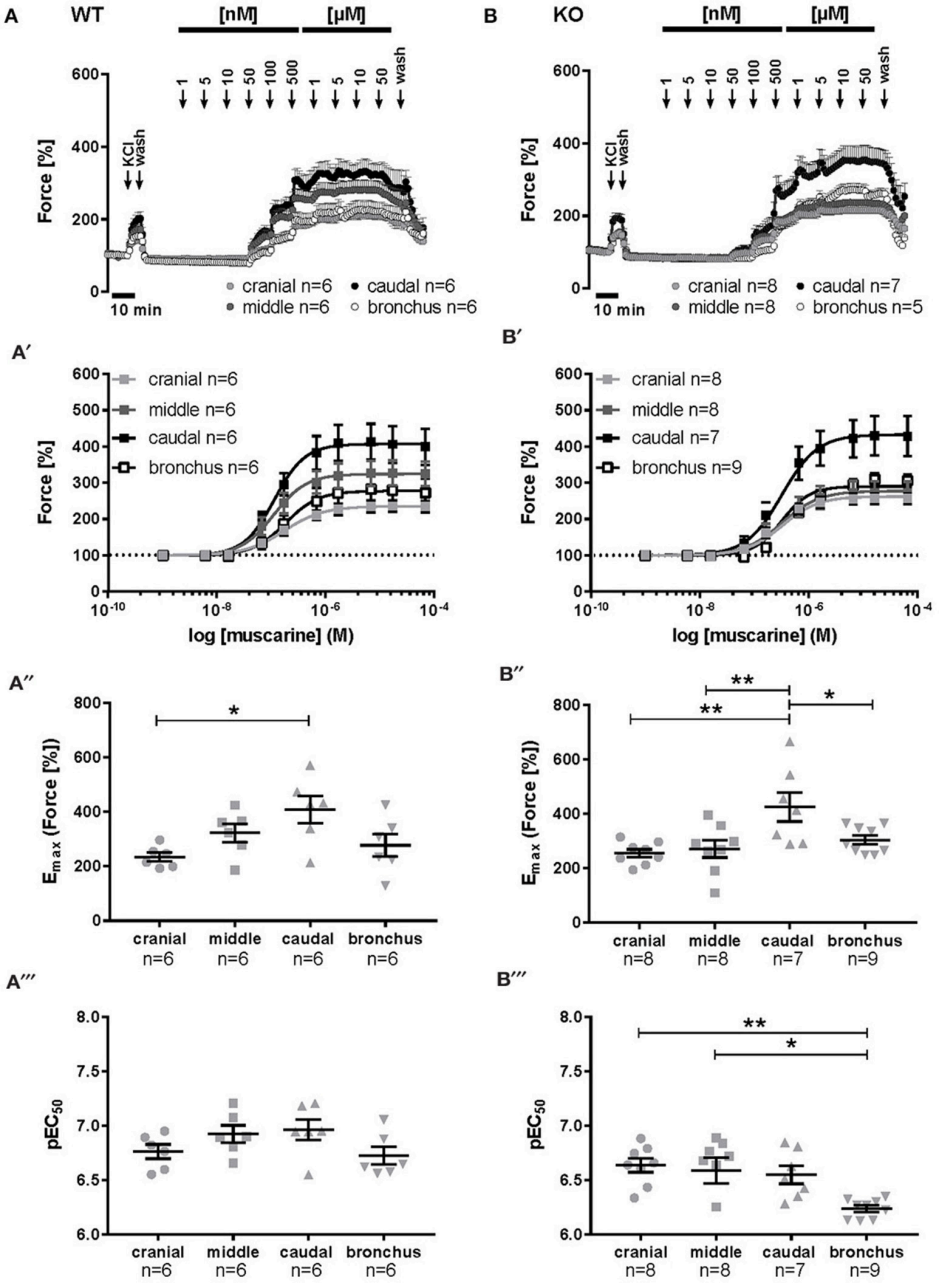
**Muscarine-mediated changes in constrictor force and reactivity in different parts of the trachea and extrapulmonary bronchi from cav-1^+/+^ and cav-1^−/−^ mice**. Organ bath recordings, each point represents the mean of number of animals (*n*) ± SEM. **(A,B)** Muscarine induces concentration-dependent contraction of ASM in cav-1^+/+^ **(A)** and cav-1^−/−^ **(B)** mouse strains. **(A',B')** Constrictor force in all parts of the airways of cav-1^+/+^ **(A')** and cav-1^−/−^ **(B')** mice. Following equilibration, baseline tension was adjusted to 0.5 g for all airway segments in all preparations. Baseline was set as 100% and the maximum response at each concentration was calculated. Sigmoidal concentration-response curves were plotted according to the Hill equation. Maximal responses (E_max_, **A”,B”**) and pEC_50_ **(A”',B”')** values for muscarine were estimated for each individual experiment. In cav-1^+/+^ the caudal part responded with a stronger contraction, whereas there was no difference in pEC_50_ values between segments. In cav-1^−/−^ the caudal part responded with a stronger contraction whereas the pEC_50_ decreased in the bronchus. ^*^p ≤ 0.05; ^**^p < 0.01, One-way ANOVA followed by Tukey's multiple comparisons test with the exception of **(B”')** which was analyzed by Kruskall-Wallis test followed by Dunn's multiple comparisons test.

Muscarine induced a dose dependent contraction in all mouse tracheal segments (Figures 3A,A'). At cumulative administration of muscarine, significantly higher potency was observed only in the caudal trachea in comparison to the cranial part (E_max_ = 391.7 vs. 231.8%, respectively, *p* ≤ 0.05) (Figure 3A”). The pEC_50_ values were not significantly affected between the tracheal parts (cranial trachea pEC_50_ = 6.76, middle trachea pEC_50_ = 6.92, caudal pEC_50_ = 6.96, main bronchus pEC_50_ = 6.72) (Figure 3A”'). In cav-1^−/−^ mice (Figures 3B,B'), muscarine-induced contraction was significantly more potent in the caudal trachea (E_max_ = 406.9%) in comparison to the cranial (E_max_ = 252.3%, *p* ≤ 0.01) and middle (E_max_ = 255.4%, *p* ≤ 0.01) trachea and to the bronchus (E_max_ = 300.4%, *p* ≤ 0.05) (Figure 3B”). The pEC_50_ values for muscarine were significantly affected (Figure 3B”'). In cav-1^−/−^ mice, muscarine had the lowest potency in inducing contraction in the main bronchus (pEC_50_ = 6.24). This response was significantly lower than that observed in the cranial trachea (pEC_50_ = 6.63, *p* ≤ 0.01) and in the middle trachea (pEC_50_ = 6.58, *p* ≤ 0.05).

**Figure 4:**
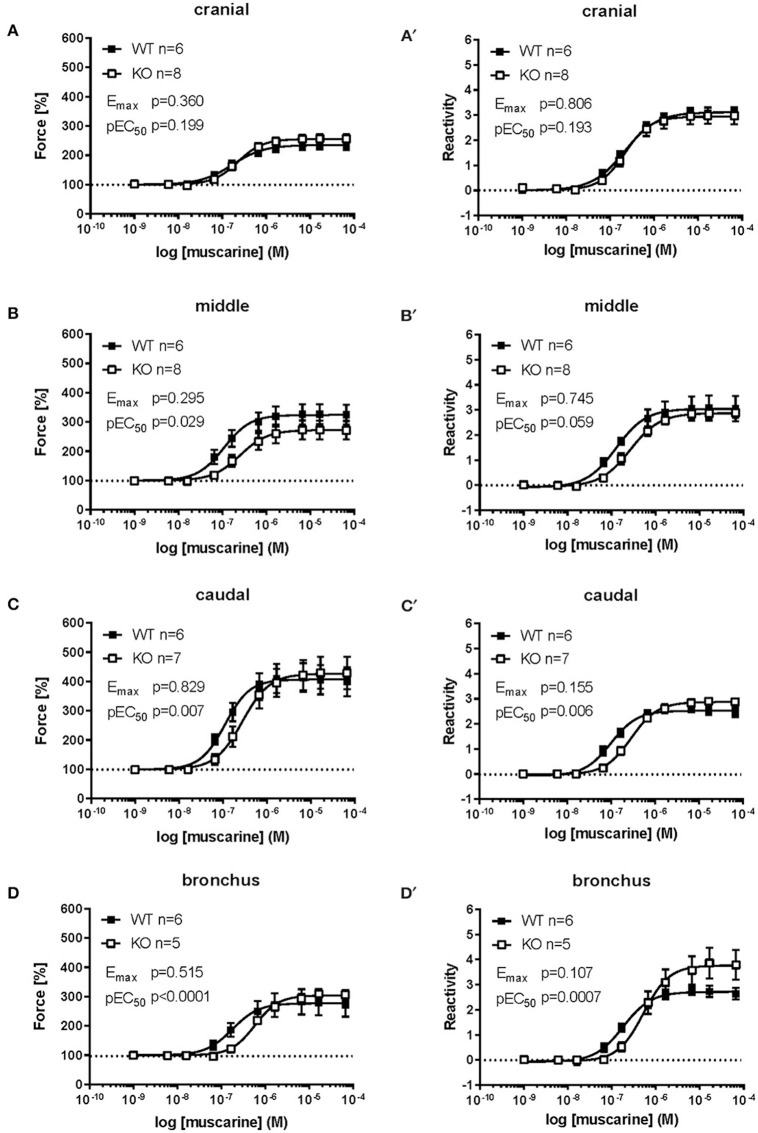
**Comparison of force and reactivity between both mouse strains**. Airway reactivity is expressed as contraction effect induced by muscarine compared to the correspondent contraction induced by KCl. Each point represents the mean of number of animals (*n*) ± SEM. Please note that when SEM is smaller than the symbol size it is not displayed in the figure. Data points were plotted as sigmoidal concentration-response curves and values for E_max_ and pEC_50_ were estimated for each individual experiment and analyzed with Student's unpaired *t*-test except for data shown in **(B,B')**, which was analyzed by Mann-Whitney *U*-test. The cranial part of the cav-1^−/−^ trachea shows no significant difference compared to cav-1^+/+^ in both force **(A)** and reactivity **(A')** levels. The middle **(B,B')** and caudal **(C,C')** part of the cav-1^+/+^ trachea were more sensitive to muscarine as indicated by a right-shift in the concentration-response curves and significant decrease in the pEC50 values for muscarine in both force and reactivity in the cav-1^−/−^ mice strain. The main bronchus **(D,D')** of cav-1^+/+^ mice was also more sensitive to muscarine compared to cav-1^−/−^ mice.

For all tracheal parts and extrapulmonary bronchi we expressed the changes in their contractile force also as changes in reactivity. At cumulative application of muscarine, contractile force and reactivity levels in the tracheal cranial part in cav-1^+/+^ mice were not significantly different from that in cav-1^−/−^ preparations (force E_max_ = 234.4 ± 16.07 vs. 255.3 ± 14.64, *p* = 0.360; reactivity E_max_ = 3.06 ± 0.20 vs. 2.95 ± 0.33, *p* = 0.806) (Figures 4A,A'). The response pattern in the middle part of the trachea was similar to that observed in the cranial trachea and the maximum efficacy did not differ between cav-1^+/+^ and cav-1^−/−^ mice (force E_max_ = 323.2 ± 33.74 vs. 271.8 ± 31.78, *p* = 0.295; reactivity E_max_ = 2.88 vs. 2.82, *p* = 0.745). A statistical comparison of pEC_50_ of the force and reactivity response in the middle tracheal part was close to a significant difference between cav-1^+/+^ or cav-1^−/−^ (for force pEC_50_ = 6.92 ± 0.08 vs. 6.59 ± 0.12, *p* = 0.029, and for reactivity pEC_50_ = 6.94 ± 0.08 vs. 6.61 ± 0.12, *p* = 0.059) (Figures 4B,B'). In the caudal trachea, no difference between cav-1^+/+^ and cav-1^−/−^ mice was observed in the maximum efficacy (force E_max_ = 409.5 ± 49.9 vs. 425.8 ± 53.52, *p* = 0.829; reactivity E_max_ = 2.54 ± 1.15 vs. 2.87 ± 0.15, *p* = 0.155) (Figures 4C,C'). However, the muscarine-induced sensitivity of the caudal trachea was significantly higher in cav-1^+/+^ mice than that observed in the corresponding cav-1^−/−^ mice (for force pEC_50_ = 6.96 ± 0.09 vs. 6.54 ± 0.08, *p* = 0.007, and for reactivity pEC_50_ = 6.98 ± 0.1 vs. 6.56 ± 0.08, *p* = 0.006) (Figures 4C,C'). Extrapulmonary bronchi of cav-1^+/+^ and cav-1^−/−^ mice showed no significant difference in E_max_ for muscarine force E_max_ = 277.6 ± 42.1 vs. 304 ± 16.6, *p* = 0.515; reactivity E_max_ = 2.72 ± 0.21 vs. 3.76 ± 0.6, *p* = 0.107) but in the pEC_50_ values (for force pEC_50_ = 6.72 ± 0.08 vs. 6.24 ± 0.03, *p* < 0.0001, and for reactivity pEC_50_ = 6.75 ± 0.08 vs. 6.27 ± 0.04, *p* = 0.0007) (Figures 4D,D').

### 5-HT-induced contraction in extra-pulmonary airway

**Figure 5:**
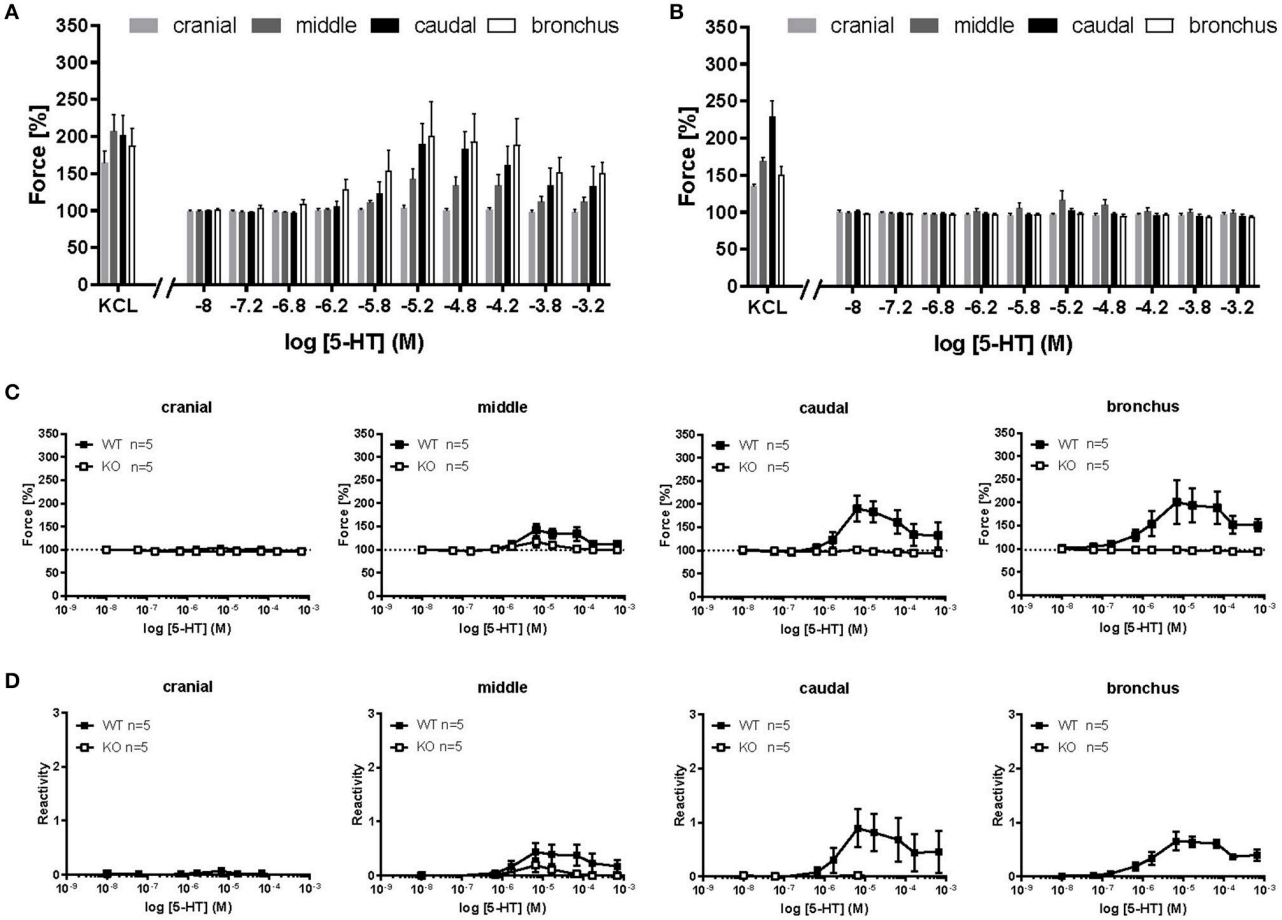
**5-HT-mediated changes in constrictor force and reactivity of different parts of the trachea and extrapulmonary bronchi from cav-1^+/+^ and cav-1^−/−^ mice**. Organ bath recordings, each point represents the mean of number of animals (*n*) ± SEM. **(A,B)** Changes in the force were recorded after cumulative application of different concentrations of 5-HT. Following equilibration, baseline tension was adjusted to 0.5 g for all airway segments in all preparations. Baseline was set as 100% and the maximum response at each concentration was evaluated. 5-HT induces concentration-dependent contraction in main bronchus, tracheal middle and caudal parts in cav-1^+/+^ mice **(A)**, whereas the cranial part of cav-1^+/+^ mice and all tracheal parts and bronchi of cav-1^−/−^ mice are not responsive to 5-HT **(A,B)**. **(C,D)** Comparison of constrictor response between both mouse strains. Note that means are not defined (and left blank) when values are 0 or negative.

The cranial part of the trachea in cav-1^+/+^ as well as in cav-1^−/−^ mice was not responding to cumulative administration of 5-HT (Figures 5A,B). In cav-1^+/+^ mice, the amplitude in the force of the 5-HT-induced contraction was concentration-dependent in the other parts of the extrapulmonary airways (Figure 5A). The response pattern of the extrapulmonary bronchi was similar to that obtained from the caudal and middle trachea (Figures 5A,C). Strikingly, the force and the reactivity response to cumulative application of 5-HT were completely abolished in extrapulmonary bronchi, in the middle and caudal trachea from cav-1^−/−^ mice (Figures 5A–D). The response to KCl (60 mM) was maintained at the end of experiments in cav-1^−/−^ mice indicating the viability of the preparations (data not shown).

### Role of cav-1 in muscarine-induced bronchoconstriction

**Figure 6:**
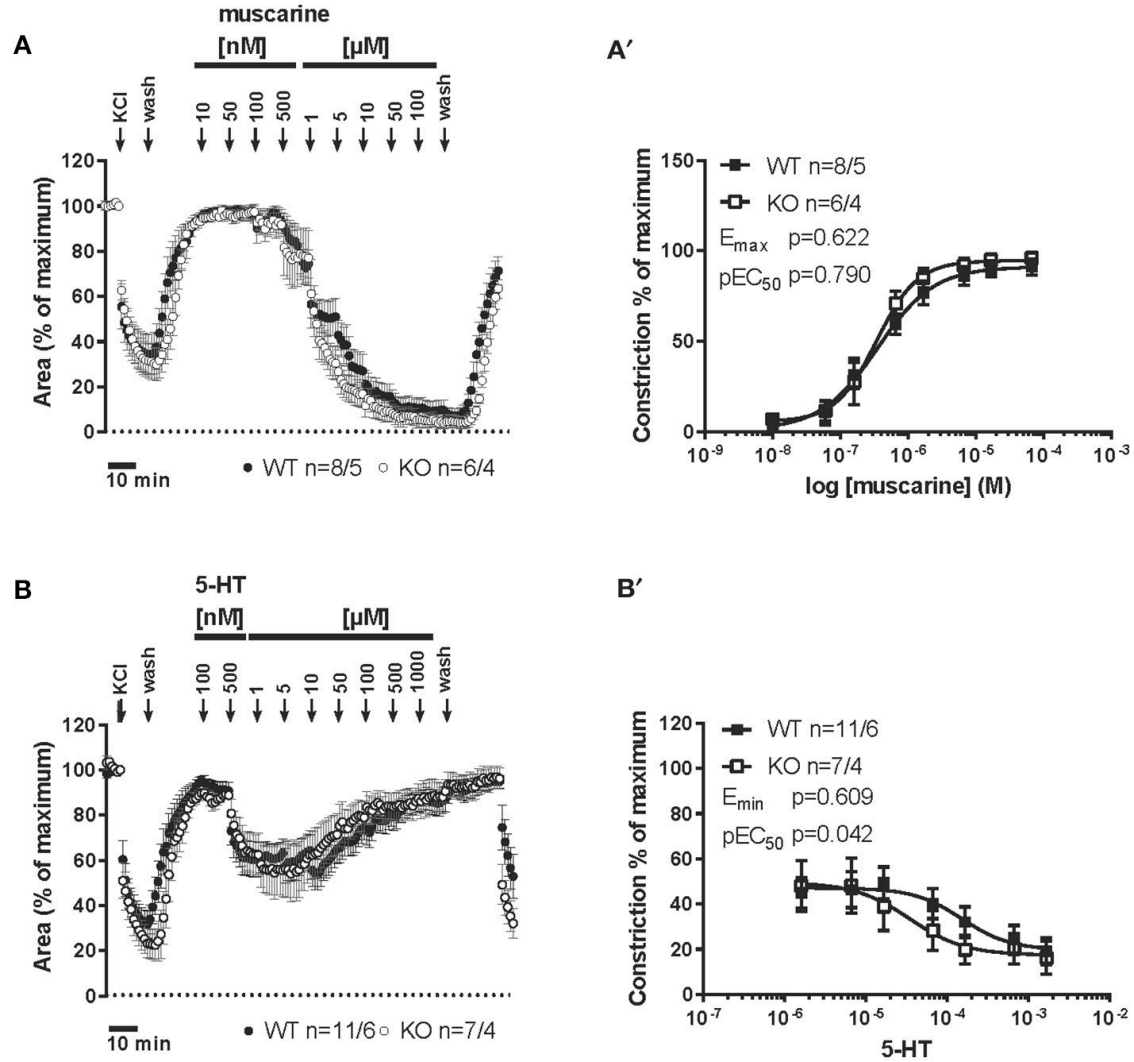
**Muscarine- and 5-HT-mediated changes in the luminal area of peripheral bronchi from cav-1^+/+^ and cav-1 ^−/−^ mice**. Videomorphometric analyses of PCLS. Data are presented as mean of number of bronchi (*n*)/number of animals ± SEM. **(A)** Muscarine induced concentration-dependent decreases in the luminal airway area in cav-1^+/+^ and cav-1^−/−^ mice. Bronchi of cav-1^+/+^ and cav-1^−/−^ mice responded to 100 nM-100 μM muscarine in a dose dependent pattern with sustained contraction. **(A')** Sigmoidal concentration-response curves of concentration vs. luminal area reduction of peripheral bronchi of cav-1^+/+^ and cav-1^−/−^ mice were plotted according to the Hill equation. Values for E_*max*_ and pEC_50_ were estimated for each individual experiment and analyzed with Student's unpaired *t*-test. **(B)** 5-HT induced concentration-dependent decreases in luminal airway area in cav-1^+/+^ and cav-1^−/−^ mice. Bronchi of cav-1^+/+^ and cav-1^−/−^ mice responded to 500 nM-1 mM 5-HT in a dose dependent pattern with sustained contraction followed by relaxation. **(B')** Sigmoidal concentration-response curves of the relaxation starting at 5 μM of peripheral bronchi of cav-1^+/+^ and cav-1^−/−^ mice were plotted according to the Hill equation. Values for E_min_ and pEC_50_ were estimated for each individual experiment and analyzed with Student's unpaired *t*-test.

Intrapulmonary bronchi from cav-1^+/+^ mice dose-dependently reacted to muscarine with reduction in their luminal area. The maximum efficacy (maximal constriction) was 97.2 ± 5.76 and the pEC_50_ value was 6.55 ± 0.14. However, depletion of cav-1 had no effect on these parameters (E_max_ = 93.57 ± 2.97, *p* = 0.228; pEC_50_ = 6.49 ± 0.14, *p* = 0.852) (Figures 6A,A').

### Role of cav-1 in 5-HT-induced intrapulmonary bronchoconstriction

**Figure 7:**
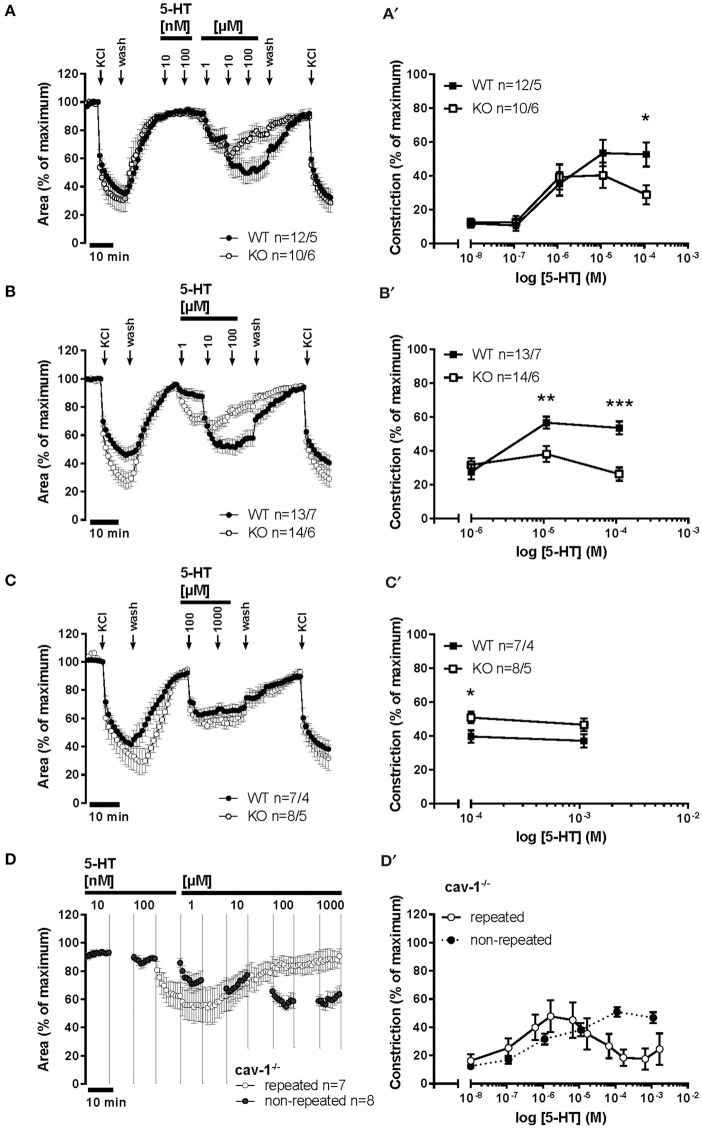
**5-HT-mediated changes in the luminal area of peripheral bronchi from cav-1^+/+^ and cav-1 ^−/−^ mice**. Videomorphometric analyses of PCLS. **(A,A')** Bronchi of cav-1^+/+^ and cav-1^−/−^ mice responded to increasing 5-HT doses. 5-HT-induced constriction in cav-1^−/−^ mice PCLS was significantly reduced at μM concentrations of 5-HT compared to cav-1^+/+^ mice strain. We applied KCl (60 mM) as a viability control for 5 min at 2 points of experiment. **(B,B')** Bronchi of cav-1^+/+^ and cav-1^−/−^ mice responded to increasing 5-HT doses. 5-HT-induced bronchoconstriction in PCLS was significantly reduced at μM concentrations in cav-1^−/−^ mice compared to cav-1^+/+^ mice. **(C,C')** Bronchi of cav-1^+/+^ and cav-1^−/−^ mice responded to 5-HT with a rapid sustained constriction. **(D,D')** Comparison of the response to 5-HT after repetitive stimulation (in half logarithmic mode) with the direct (non-repetitive) 5-HT response. The intrapulmonary bronchi of cav-1^−/−^ mice exhibit a decrease in 5-HT-induced bronchoconstriction after repetitive 5-HT application. Data are presented as mean of number of bronchi (*n*)/number of animals ± SEM. ^*^p ≤ 0.05; ^**^, *p* < 0.01; ^***^p < 0.001, Student's unpaired *t*-test with the exception of values at 1 mM 5-HT in **(C')** which was analyzed by Mann-Whitney *U*-test.

Intrapulmonary bronchi from cav-1^−/−^ and cav-1^+/+^ mice showed the same pattern of response to stimulation with gradually increasing doses (100 nM-1 mM in half logarithmic mode) of 5-HT. The response to 5-HT started with 100 nM (Figure 6B) and the maximum constrictor response was observed at 5 μM in cav-1^+/+^ as well in cav-1^−/−^ mice. This maximal constrictor response was 44.99 ± 6.69 in cav-1^+/+^ mice and 48.1 ± 11.27 in cav-1^−/−^ mice (*p* = 0.803, Student's unpaired *t*-test). At higher concentrations, the cumulative application of 5-HT caused a concentration-dependent transient relaxation in both, cav-1^−/−^ and cav-1^+/+^ mouse strains. We analyzed this relaxation according to a non-linear regression and determined values for maximal relaxation (termed E_min_ for clarity) and sensitivity (pEC_50_). There was no significant difference in values for E_min_, however, the 5-HT-induced sensitivity was significantly smaller in cav-1^+/+^ mice than that observed in the corresponding cav-1^−/−^ mice (pEC_50_ = 3.47 ± 0.45 vs. 4.55 ± 0.15, *p* = 0.042; Figure 6B'). To address the possibility that 5-HT receptors might be sensitized or desensitized after prolonged or repeated application of 5-HT, we used different 5-HT application modes. When the bronchi were repetitively stimulated with cumulative doses of 10 nM-100 μM 5-HT, the maximal constrictor response was observed at 10 μM in cav-1^+/+^ as well in cav-1^−/−^ mice (Figures 7A,A'). Interestingly, the cav-1-deficient bronchi had a significantly weaker bronchoconstrictor response to 5-HT at 100 μM (*p* = 0.021) (Figures 7A,A'). Next, we performed less repetitive 5-HT stimulation (1 μM-100 μM) on intrapulmonary bronchi (Figures 7B,B'). The absence of cav-1 led to a significant decrease in 10 μM (*p* = 0.004) and 100 μM (*p* < 0.0001) 5-HT-induced bronchoconstriction. Last, we started stimulation with 5-HT at 100 μM followed by application of a supramaximum dose of 5-HT (1 mM). Intrapulmonary bronchi responded to 100 μM 5-HT with a maximum constrictor response for 5-HT and the deletion of cav-1 marginally increased the effect of 5-HT at 100 μM (*p* = 0.045) (Figures 7C,C'). Next, we compared repetitive 5-HT stimulation (100 nM-1 mM in half logarithmic mode) and the response to the same 5-HT-dose without cumulative stimulation (10 nM from cumulative doses of 10 nM-100 μM; 100 nM from 100 nM-1 mM; 1 μM and 10 μM from 1 μM-100 μM; 100 μM and 1 mM from 100 μM-1 mM). The contractor response to repeated 5-HT stimulation was shifted to the left meaning that repetitive stimulation with low doses of 5-HT (nM) leads to stronger constriction and at higher doses to enhanced relaxation. Interestingly, the maximum response was not affected in both cases (Figures 7D,D').

### Effect of MCD-treatment on muscarine and 5-HT-induced bronchoconstriction

**Figure 8:**
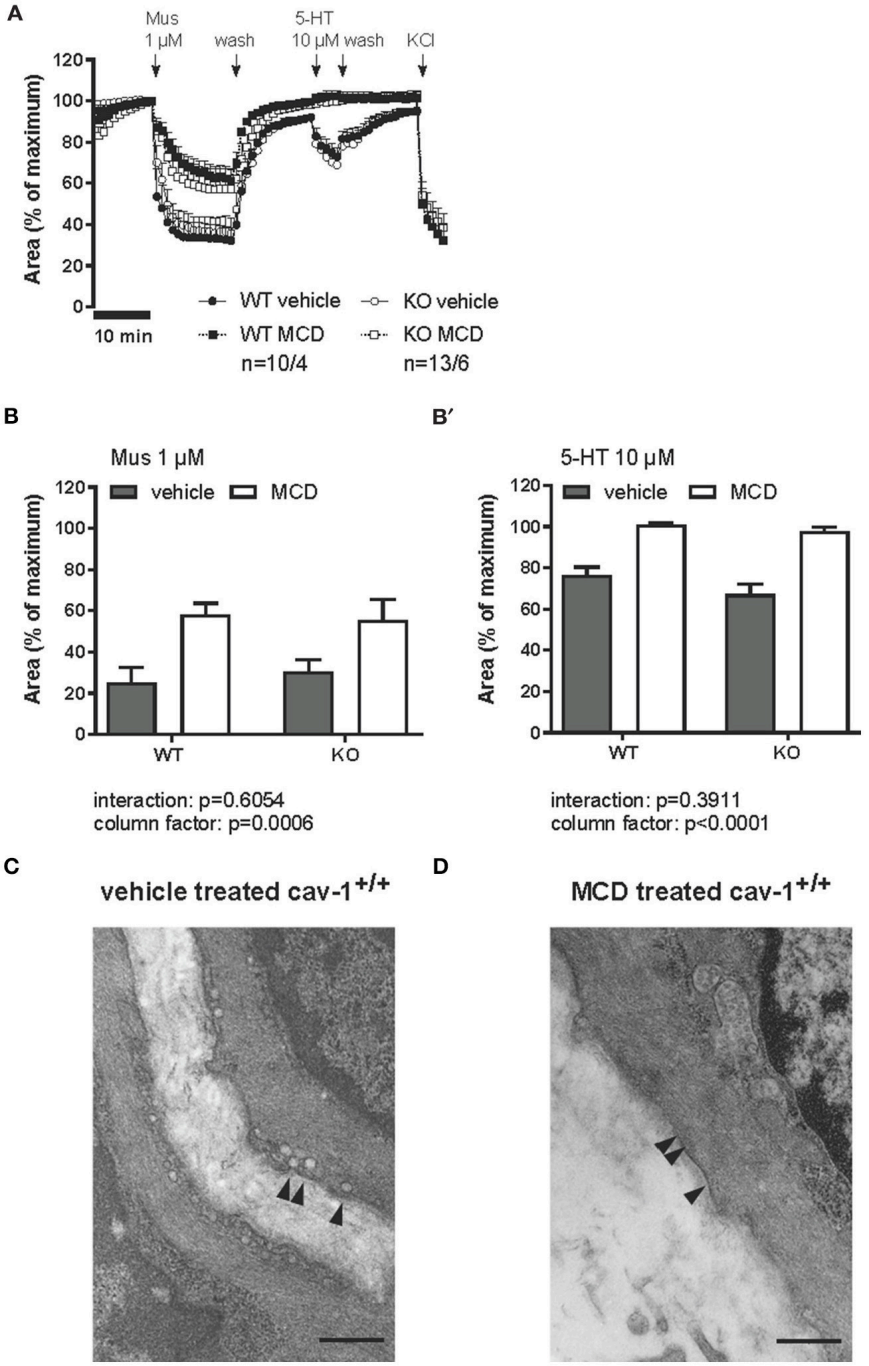
**Muscarine- and 5-HT-mediated changes in the luminal area of peripheral bronchi from cav-1^+/+^ and cav-1^−/−^ mice after vehicle (^___^) or MCD (- - - -) treatment**. Changes in the luminal area of mouse peripheral airways were recorded by videomorphometry after application of 1 μM muscarine (Mus), 10 μM 5-HT and 60 mM KCl for vehicle (^___^) or MCD (- - - -) treatment. Data are presented as means ± SEM; *n* = number of bronchi/animals with baseline value set as 100%. **(A)** The bronchi from cav-1^+/+^ and cav-1^−/−^ mice constrict in response to Mus and 5-HT. In both mouse strains, caveolae disruption by MCD reduces the response to Mus whereas the response to 5-HT is fully abrogated. No differences in the response to KCl occur after MCD-treatment in either mouse strain. **(B)** Bar graphs of the maximum response after application of 1 μM Mus and 10 μM 5-HT. Luminal area of peripheral bronchi of cav-1^+/+^ and cav-1^−/−^ mice after vehicle (gray) or MCD (white) treatment. Data was analyzed by 2-way ANOVA. Whereas the *p*-value of the column factor indicates that MCD is effective in both mouse strains, there is no statistically significant interaction. **(C)** Transmission electron microscopy of intrapulmonary bronchi derived from PCLS included in videomorphometric experiments. Vehicle-treated murine ASM containing areas with caveolae (arrowheads) in the plasma membrane of cav-1^+/+^ mice. **(D)** Cell surface region of an equivalent bronchial SM after caveolae disruption by MCD. Arrows point to plasma membrane without caveolae and bar = 500 nm.

Intrapulmonary bronchi from cav-1^+/+^ and cav-1^−/−^ mice constricted strongly in response to 1 μM muscarine and to 10 μM 5-HT (Figure 8). The magnitude of the muscarine-induced bronchoconstriction was higher than constriction evoked by 5-HT (Figure 8A). In order to determine if caveolae are implicated in the contractile response to muscarine and 5-HT, we treated PCLS with known contractile response from both cav-1^+/+^ and cav-1^−/−^ mice by 1 μM MCD. The KCl response was not altered after MCD-treatment. The bronchoconstrictor responses of the PCLS were unchanged after vehicle treatment in both mouse strains. In contrast to untreated bronchi, incubation with MCD in cav-1^+/+^ as well as in cav-1^−/−^ mice partially inhibited the 1 μM muscarine-induced constriction (column factor of *p* = 0.0006, analyzed by 2-way ANOVA) (Figure 8B). The same concentration of MCD had a pronounced effect on 5-HT-induced constriction. Intrapulmonary bronchi were entirely unresponsive to 10 μM 5-HT in cav-1^+/+^ and cav-1^−/−^ mice (column factor of *p* < 0.0001, analyzed by 2-way ANOVA) (Figure 8B). There were no significant interactions between MCD treatment and mouse strains of the responses to muscarine (*p* = 0.605) and 5-HT (*p* = 0.391; both analyzed by 2-way ANOVA). Interestingly, we found high numbers of caveolae in ASM from vehicle-treated PCLS. Caveolae were arranged mostly side by side in rows (Figure 8C). In PCLS treated with MCD, caveolae were completely absent from ASM (Figure 8C).

### 5-HT receptor subtypes mRNA expression

**Figure 9:**
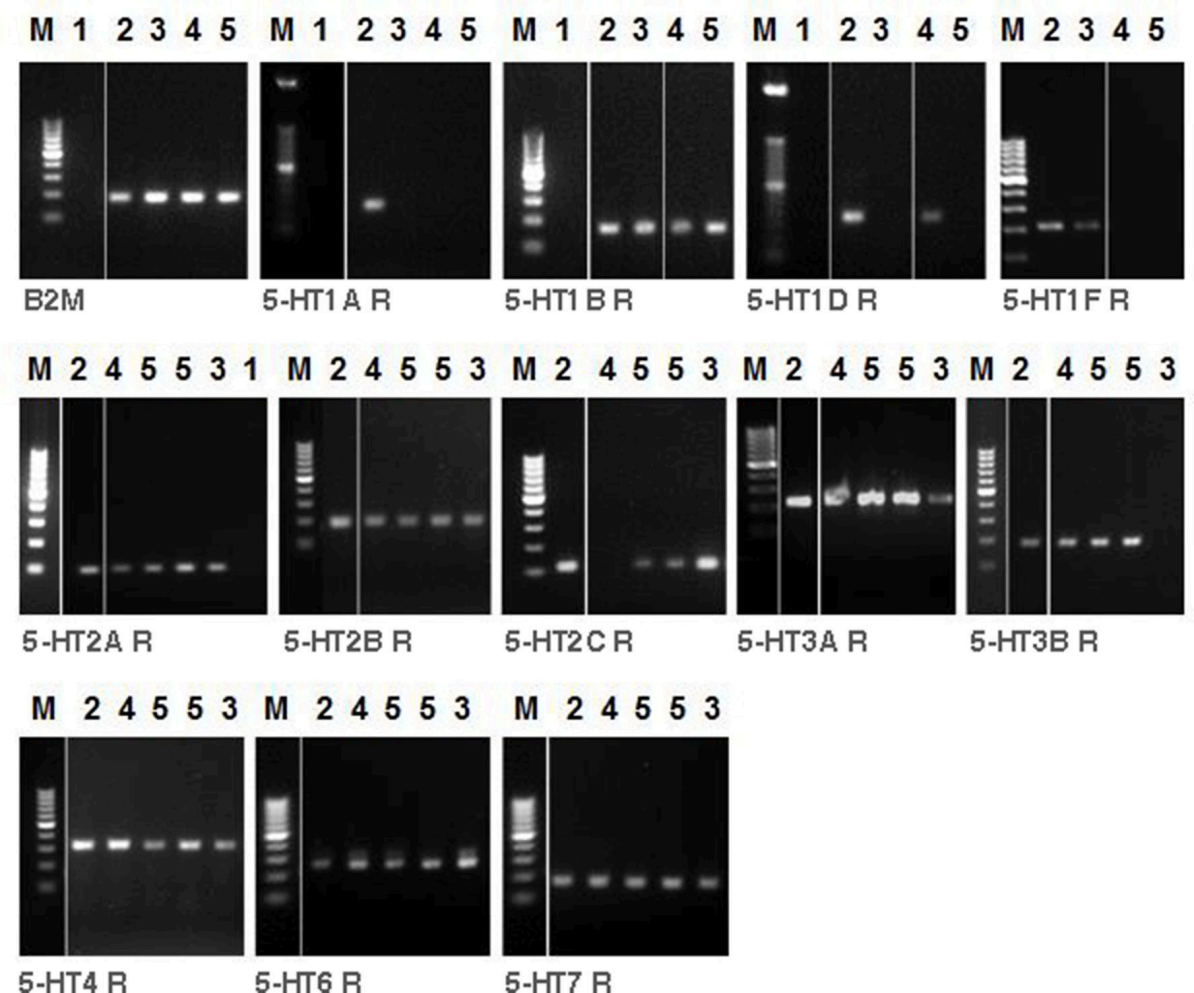
**RT-PCR analysis of 5-HT receptor subtypes in tracheal muscle, main bronchus and lung homogenate of C57BL/6J mice**. The presence of mRNA coding for 5-HT receptor subtypes are shown. *M* = marker. ß-2-Microglobulin (B2M) as housekeeping gene control was run for different samples. H_2_O (1) as control reactions included the absence of template, brain (2) as positive control, lung (3), tracheal muscle (4) and main bronchus (5).

In order to determine which type of 5-HT receptors are implicated in the contractile response to 5-HT in the airways, we investigated the expression level of all 5-HT receptors in the tracheal muscle, bronchus and lung of C57BL/6J mice. Tracheal muscle, bronchial and lung homogenates showed different patterns of 5-HT receptor subtypes expression (Table 2). No mRNA was detected for 5-HT1A in tracheal muscle of 4 tested samples. The most prevalent subunits in the airways were 5-HT1B, 5-HT2A, 5-HT6, and 5-HT7. No bands were detected in control reactions when no DNA template was present (Figure 9).

**Table 2:** **Expression of different 5-HT receptor subtypes in the murine airway**.

**5-HT receptor**	**5-HT1A**	**5-HT1B**	**5-HT1D**	**5-HT1F**	**5-HT2A**	**5-HT2B**	**5-HT2C**	**5-HT3A**	**5-HT3B**	**5-HT4**	**5-HT6**	**5-HT7**
Tracheal muscle	0	4	4	3	4	3	2	3	4	4	4	4
Main bronchus	1	4	3	2	4	4	2	3	4	4	4	4
Lung	2	4	2	2	4	4	3	2	1	2	4	4

*Numbers represent positive samples out of four tested mice*.

### Effect of the 5-HT2A antagonist, ketanserin, on 5-HT induced bronchoconstriction

**Figure 10:**
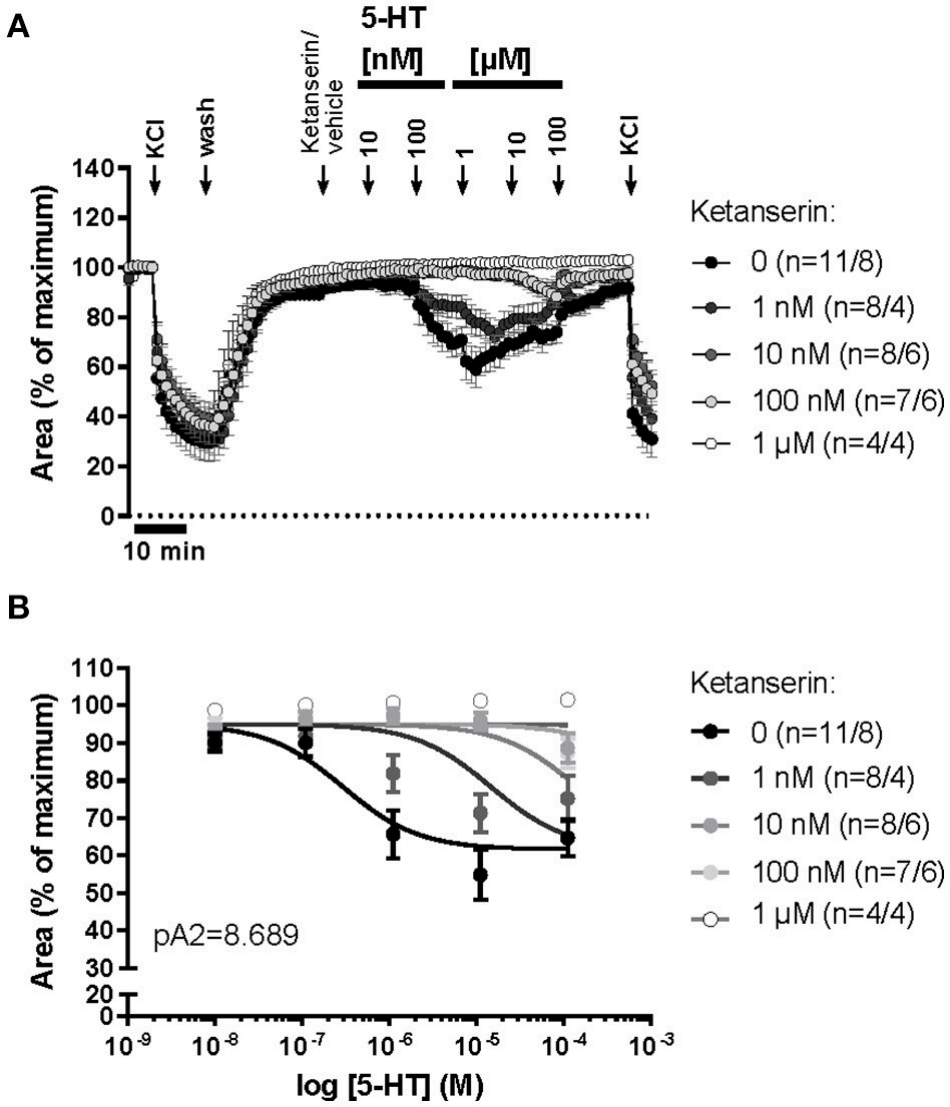
**5-HT-mediated changes in the luminal area of peripheral bronchi from cav-1^−/−^ mice after treatment with vehicle or with the 5-HT2A receptor antagonist, ketanserin. (A)** Changes in the luminal area of mouse peripheral airways was recorded by videomorphometry in PCLS after cumulative application of different concentrations of 5-HT and KCl. 5-HT induces a decrease in luminal airway area in cav-1^−/−^ mice. **(B)** Sigmoidal concentration-response curves for 5-HT were plotted according to the Hill equation in the presence of the different doses of ketanserin and the pA2-value for ketanserin (8.689) was estimated by Schild-analysis. Hill-slope and Schild-slope were fixed to 1.0. Data are presented as mean of number of animals (*n*) ± SEM.

In order to evaluate the role of the 5-HT2A receptor subtype in 5-HT-induced constriction of intrapulmonary murine airways, we measured the contractile response to ascending concentrations of 100 nM-100 μM 5-HT before and after treatment with a single dose of ketanserin (1 nM, 10 nM, 100 nM, or 1 μM) as a high-affinity antagonist of the 5-HT2A receptor subtype. In cav-1^−/−^ mice, ketanserin (10 nM and 100 nM) inhibited the 5-HT-induced constriction with a pA2 of 8.689 (Figures 10A,B) while the response to KCl was fully maintained (Figure 10A).

## Discussion

The present data show that the effects of cav-1 deficiency on regulation of airway tone are agonist-specific and dependent on airway level. Cholinergic (muscarinic) responses were modestly modified and included in diminished agonist sensitivity (lower pEC_50_ values) in the middle and caudal trachea, and in the extrapulmonary bronchi. Using a similar methodological approach, i.e., force recording from tracheal segments containing three to four cartilage rings, Sharma et al. have not noted a difference in sensitivity to methacholine between cav-1^−/−^ and cav-1^+/+^ tracheae (Sharma et al., 2012). In this study, however, segments from all tracheal levels had been pooled, which might have had masked effects that do not occur along the entire length of the trachea, as they have been demonstrated in the present study. In our preparation, the different tracheal segments had comparable size and therefore we assume that the amount of the smooth muscle was not varying much. Supportively, we did not observe any differences in pEC50 values for muscarine in cav-1^+/+^ mice and in the responses to 5-HT in cav-1^−/−^ mice in the trachea. Indeed, cav-1 deficiency did not cause changes in the potency of muscarine to induce contraction in the cranial tracheal part, but a decrease toward the caudal segments of the extrapulmonary airways as indicated by the right shift in the concentration response curves. We cannot exclude that differences in the amount of the smooth muscle could be present between the tracheal segments, the extrapulmonary bronchi and the intrapulmonary bronchi in our preparation. Whether the differences in contractile responses that we have observed in cav-1^+/+^ mice were due to variation in the amount of the smooth muscle in different locations of the airway tree or to variation in excitation-contraction coupling is very intriguing and needs to be addressed in future studies. Although, several links of cav-1 to muscarinic receptor signaling have been reported at the cellular level in ASM (Gosens et al., 2007; Prakash et al., 2007; Sharma et al., 2010), they appear not to be essential for cholinergic constriction *per se* but rather modulate the sensitivity of the system. Silencing of cav-1 expression in tracheal smooth muscle cells disrupted the direct interaction of caveolin-1 with β-dystroglycan and alters the agonist-induced intracellular Ca^2+^ release and the force generation (Sharma et al., 2010). Interestingly, although the sensitivity for muscarinic M3-receptor-mediated [Ca^2+^]_i_ mobilization was reduced, the maximum peak [Ca^2+^]_i_ remained unchanged. Supportively, loss of dystrophin reduced the sensitivity of mouse tracheal rings at submaximal concentrations of MCh but did not affect the maximal contractile response (Sharma et al., 2014). The right shift in the concentration response curves to muscarine observed in our study might be due to the disruption of the cav-1-distrophin-complex. Indeed, this even applies to the entire structural caveolar complex since cav-1 deficiency resulted in an almost entire loss of ultrastructurally discernible caveolae in the ASM plasma membrane. The few remaining caveolae, as they have also been reported in vascular and ileal smooth muscle cells in cav-1^−/−^ mice (Gherghiceanu et al., 2009; Cipriani et al., 2011), are probably organized by cav-3 which is additionally expressed in smooth muscle cells (Schlenz et al., 2010). These data demonstrate that cholinergic bronchoconstriction either does not require a caveolar signaling platform (Sharma et al., 2010), or very few caveolae are sufficient to exert nearly the full effect.

This issue was further addressed by getting rid of all caveolae by treatment with the cholesterol depleting agent, MCD. In accordance with previous data (Schlenz et al., 2010), this intervention markedly reduced cholinergic bronchonstriction in PCLS. It remains to be determined whether the stronger effect of MCD-treatment compared to that of cav-1 deficiency is due to the loss of the remaining population of caveolae or to disruption of non-caveolar cholesterol-rich plasma domains. In any case, approximately 50% of the constrictor response was maintained after MCD-treatment in both cav-1^+/+^ and cav-1^−/−^ mice, demonstrating that at least a large extent of cholinergic bronchoconstriction is initiated in non-caveolar plasma domains. In support of this conclusion, MCD-treatment reduced sensitivity but not maximum isometric force induced by ACh in canine tracheal muscle strips (Gosens et al., 2007).

Much in contrast, serotonergic bronchoconstriction recorded in PCLS was fully abrogated after depletion of cholesterol from the plasma membrane, as already reported earlier (Schlenz et al., 2010), demonstrating that it is to a large extent orchestrated in plasma domains principally different from those linking muscarinic receptors to bronchoconstriction. Along the same line of argumentation as for muscarinic bronchoconstriction, this MCD-sensitive plasma domain is not a structural caveola as serotonergic bronchoconstriction was maintained in near absence of caveolae caused by lack of cav-1. As for muscarinic receptors, cav-1 rather organizes a modulation of than being crucial for serotonergic bronchoconstriction. Specifically, the desensitizing effect which we observed at repetitive stimulation with increasing doses of 5-HT was more marked in bronchi from cav-1^−/−^ mice.

Serotonergic constriction of intrapulmonary bronchi is considered to result from direct activation of 5-HT receptors located on ASM (Kummer et al., 2006). In the present study, this effect was sensitive to ketanserine blockade of the 5-HT2A receptor, one of the receptors consistently found to be expressed by RT-PCR analysis. This receptor is also associated with serotonergic bronchoconstriction in other species, although other subtypes may be additionally involved (Van Nueten et al., 1982; Tolloczko et al., 1995; Cazzola and Matera, 2000; Segura et al., 2010). Accordingly, administration of a 5-HT2A antagonist and a 5-HT1A agonist were effective in patients with status asthmaticus and obstructive airway disease, respectively (Prezant and Aldrich, 1988; Cazzola et al., 1990). 5-HT2A has been linked to caveolins and caveolar signaling, but with marked cell type specific differences. In glioma cells and pulmonary artery smooth muscle cells, the 5-HT2A receptor co-immunoprecipitates with cav-1 (Bhatnagar et al., 2004; Cogolludo et al., 2006), and in cardiomyocytes with cav-3 (Mialet-Perez et al., 2012). The only study addressing its coupling in ASM, however, reports an independence from cav-1 and restriction to non-caveolar membrane fractions in bovine tracheal smooth muscle (Sommer et al., 2009). This is consistent with the present finding of grossly maintained bronchoconstrictor responses in absence of cav-1 and caveolae.

In contrast to the direct constrictor effect upon ASM of murine intrapulmonary bronchi, serotonergic contraction of the murine trachea is assumed to be an indirect effect based on triggering of acetylcholine release of either nerve fibers or epithelial cells (Eum et al., 1999; Moffatt et al., 2004). This is likely to explain the fundamental difference in the consequence of cav-1 deficiency we have observed between intrapulmonary bronchi and extrapulmonary airways, which entirely lost 5-HT induced muscle contraction. Since muscarinic contraction of trachea and main bronchus was not attenuated in cav-1^−/−^ mice, the cholinergic component of this indirect effect is not responsible for the abrogated response to serotonin. Among the potential candidates releasing acetylcholine upon stimulation with 5-HT, i.e., epithelial cells and cholinergic nerve fibers, only basal epithelial cells displayed cav-1-immunoreactivity, consistent with our earlier observations (Krasteva et al., 2006). Notably, basal cells do not extend into intrapulmonary bronchi in mice, which correlated with the maintained and direct serotonergic effect at this airway level in cav-1^−/−^ mice.

Collectively, the roles of cav-1, caveolae, and cholesterol-rich plasma domains in regulation of airway tone are highly agonist-specific and dependent on airway level. Cav-1 is indispensable for serotonergic contraction of murine trachea and main bronchus, which is generally considered to be indirectly mediated through acetylcholine release from nerve fibers or epithelial cells. Those serotonergic and cholinergic effects which are considered to result from direct activation of ASM, i.e., intrapulmonary bronchoconstriction and cholinergic contraction of extrapulmonary airways, are largely maintained both in cav-1 deficiency and in near absence of structural caveolae. Still, cav-1/caveolae are involved in modulatory processes at this level, which shall be considered in settings such as bronchial hyperreactivity being associated with common airway diseases. Since cholesterol depletion from the membrane differentially affected cholinergic (partial loss) and serotonergic (full abrogation) bronchoconstriction, these two bronchoconstrictor pathways appear to be initiated from distinct membrane compartments. In conclusion, cholinergic and serotonergic ASM constrictor pathways do not only differ in their obviously specific initial receptors, but also in their coupling to cav-1 and their plasma membrane organization, and appropriate intervention at this post-receptor level allows for selective interference with ASM tone ranging from modulation of agonist sensitivity to full inhibition of bronchoconstriction.

In conclusion, our present study identifies cav-1 as a member of the caveolar coat complex in the airways which determines specific functions in this signaling platform albeit being dispensable for structural maintenance of this compartment. Its role in regulating cholinergic airway sensitivity is restricted to middle and caudal parts of trachea. In contrast to its rather subtle role in modulation of cholinergic bronchoconstriction, cav-1 is crucial for serotonergic contraction of extrapulmonary airways, where it is also expressed by basal epithelial cells. These epithelial cells are assumed to mediate serotonergic contraction of the trachea. The absence of cav-1 from epithelial cells of intrapulmonary bronchi correlates with unaltered serotonergic response of these airways in cav-1^−/−^ mice. Further studies on cav-3^−/−^ mice are also needed for elucidating the compensatory role of caveolins in SM bronchoconstriction. Potentially, these data warrant consideration during pharmacological modulation of the cholinergic and serotonergic responses and provide an opportunity to modulate airway hyperreactivity.

## Author contributions

GK designed the study and interpreted the data, HS performed the videomorphometry recordings and electron microscopical analyses, MS performed electron microscopy, PH and SW performed the organ-bath recordings and analyses, PH performed the RT-PCR. MK and MA analyzed and interpreted the data. MK performed the histological examinations. GK, WK, and MK wrote the manuscript. All authors read and approved the final manuscript.

## Funding

This work was funded by the Excellence Cluster Cardio-Pulmonary System to GK, by the Deutsche Forschungsgemeinschaft (DFG): Transregional Collaborative Research Center 84 to GK and WK, and by the GRK 1062 Signaling Mechanisms in Lung Physiology and Disease (SMLPD) to WK.

### Conflict of interest statement

The authors declare that the research was conducted in the absence of any commercial or financial relationships that could be construed as a potential conflict of interest.

## Abbreviations

Cav-1: caveolin-1
RT-PCR: reverse transcription-polymerase chain reaction
ASM: airway smooth muscle
SEM: standard error of mean
MCD: methyl-beta-cyclodextrin
5-HT: 5-hydroxytryptamine
KO: knockout
WT: wildtype
Emax: maximum efficacy
pEC50: half maximal effective concentration expressed as a negative logarithm of EC50, Mus, muscarine.
